# High occurrence of perianal abscess among Bedouin compared to Jews in the southern region of Israel

**DOI:** 10.1186/1471-2482-13-35

**Published:** 2013-09-12

**Authors:** David Czeiger, Gad Shaked, Igor Igov, Ilia Pinsk, Jochanan Peiser, Gilbert Sebbag

**Affiliations:** 1 Department of Surgery, Soroka University Medical Center and Ben-Gurion University of the Negev, Beer-Sheva, Israel; 2 Laniado Medical Center, Netanya, Israel; 3 Department of Surgery B, Soroka University Medical Center, PO Box 151, Beer-Sheva, Israel

## Abstract

**Background:**

This study assessed the ethnic differences of perianal abscess between Bedouin and the general population in southern region of Israel. Israeli-born Arabs have much less colorectal cancer than Israeli-born Jews. It is not clear whether other colorectal diseases have the same ethnic occurrence.

**Method:**

This is a retrospective case series of patients who had perianal abscess. Patients' demographics, managements and course of disease were analyzed.

**Results:**

Bedouin male constituted 29.7% of all patients, while they constitute only 15.7% of the population relative risk of 2.27 *(p*< 0. 001). 16.4% of the patients experienced perianal abscess recurrence. 39% of the males with recurrent abscess formation were Bedouin, relative risk of 1.8 (*p*<0. 001).

**Conclusion:**

Bedouin males have high relative risk to develop perianal abscess. Bedouin males as others with first recurrence have high relative risk for recurrence. Thus for both groups of patients, there is an indication to operate in order to treat the abscess and coexisting fistula.

## Background

Clinical observations in our medical center led us to believe that there are ethnic differences among patients operated for perianal infectious diseases. It is known that the incidence of colorectal cancer (CRC) and Inflammatory Bowel Diseases (IBD) is significantly lower among Arabs in Israel than among both Israeli-born and immigrant Jews [1]. The purpose of the present study is to assess whether perianal abscess reflect ethnic differences between Bedouin and the general population in the Negev region of Israel. We explored the occurrence, treatment and outcome of perianal abscess in patients at Soroka University Medical Center (SUMC), a 1000 bed hospital located in the Negev region of Southern Israel. The SUMC deserves nearly 500,000 inhabitants. Bedouin Arabs constitute the largest non-Jewish ethnic group in the area.

Perianal abscess is defined as a collection of pus in the perianal tissues. Abscesses of the perianal region are the most common proctological disorder requiring immediate surgery and are a frequent reason for surgery in hospitals [2]. Anorectal abscesses are mainly the result of cryptoglandular infections [3]. The obstruction of these glands ducts results in stasis and suppuration, which may lead to the development of an intersphincteric abscess. The pus formed is unable to drain into the anal lumen; caudal spread is the simplest and most frequent way in which these infections disseminate. Prompt diagnosis and surgical drainage of the abscess are required to avoid further spreading of the infection which can lead to formation of abdominal retroperitoneal abscess, Fournier's gangrene, and in rare cases, systemic infection. Due to the importance of immediate surgery and the fact that patients may well arrive at the emergency room (ER) when experienced proctologists are not available, patients are mostly treated by residents or general surgeons.

## Methods

We reviewed the records of all our institution inpatients diagnosed in the emergency room and treated for perianal abscess during an 11 year period. The local institutional ethics committee of Soroka university medical center reviewed and approved the study (Approval number 10600). Data collected from the institutional medical records included demographic details, background concomitant health conditions such as diabetes mellitus, obesity, and cigarette smoking, type of treatment, and outcome. We extracted the demographic composition of Israel's southern region from the National Bureau of Statistics [4] and compared the incidence of perianal abscess among the Bedouin population to the rest of the population in our region. We also calculated the rate of recurrence (once or multiple) in the two groups.

### Statistical analysis

The statistical analysis was done with GraphPad Prism® version 4.0 for the Health Sciences (San Diego, CA). Statistical comparison was performed using the chi-square test with Yates correction and Fisher’s exact test. P< 0.05 was considered significant. Risk was calculated as relative risk (RR) with confidence interval (CI) of 95%.

## Results

During the 11 year span of this study, 1415 incision and drainage (ID) procedures were performed on 1144 patients (Table 1). Eighty percent of the operations were performed on males, 188 patients needed at least one additional ID due to recurrence of perianal abscess; overall, 271 procedures were performed in addition to the original ID.

**Table 1:** Occurrence of "Incision and Drainage" operation for perianal abscess, among Bedouin and Jews during 11 years period

	**Bedouin**	**General population**	**Total**
Female	36(12.8%)	247(87.2%)	283
Male	337(29.8%)	795(70.2%)	1132
Total	373	1042	1415

Bedouin constituted 23.2% of all patients, 89.5% of them were males (N=236). Females represented 10.5% (N=29) of the Bedouin patients. In contrast, in the Jewish population, males represented only 76% (N=668), and the females 24% (N=211). The Bedouin patients males and females were significantly younger than the general population (Table 2). The overall hospital length of stay was 2.1±0.8 days.

**Table 2:** The mean age of patients

	**General population**	**Bedouin**
Female	36.55±10.82*	42.97±15.57*
Male	35.75 ±12.66&	44.35±14.12&

* And & marks groups with statistically significant difference (P< 0.001).

In the Negev region of Israel, adult Bedouin males constitute only 15.7% (Table 3) of all males [4]. But Bedouin males had 29.8% of the incision and drainage of perianal abscesses, a significantly higher proportion (p< 0.0001). In contrast, only 12.8% of the procedures in female were done in Bedouin female patients, similar to their proportion in the general population.

**Table 3:** Demographic composition of adult’s in the Beer- Sheva region in 2005

	**Bedouins**	**General population**	**Total**
Female	24.8(15.2%)	138.4(84.2%)	163.2
Male	24.3(15.7%)	130.5(84.2%)	154.8
Total	49.1	268.9	318

Data expressed in thousands.

One hundred eighth eight (16.4%) patients that underwent primary incision and drainage experienced perianal abscess recurrence, 41 (21.8%) of them had more than one recurrence. In total 271 (19.2%) operations were performed for these recurrences. Ethnic differences were also encountered with respect to recurrences: 39% of the males with recurrent abscess formation were Bedouin although they were only 29% of the male patients undergoing the primary procedure, RR of 1.8 (95% CI 1.366 to 2.418) p<0.0001. In contrast, the RR for recurrence in Bedouin females was as expected in the general population (RR of 1.34 (95% CI 0.5634 to 3.222). Twenty four patients had 2 recurrences, 11 had 3 recurrences and 6 had 4 or more. Having one recurrence further doubled the chance of developing another recurrence of a perianal abscess (95% CI 1.607 to 2.932). None of the patients having recurrence suffered from inflammatory bowel disease.

## Discussion

This study focused on differences in the ethnical pattern of perianal abscess as well as its treatment in the Israel Negev population during an 11 year period. Eighty percent of the operations were performed on males, an observation consistent with previous findings in western countries [5]. This male gender predominance in perianal abscess has been reported also for other diseases of the lower gastrointestinal tract in different populations of Bedouin; however, the pathology of perianal abscess did not receive particular consideration [6]. The mean age of the Bedouin (males and females) who underwent surgical intervention was 8 years younger than the general population (p<0.001).

Ethnic differences between Israelis inhabiting same environment have been described for colorectal cancer in which the incidence of colorectal cancer among Arabs is 10 times lower than in Jews [1]. In contrast, we found that Bedouin males had more than twice relative risk (RR 2.27 95% CI 2.002 to 2.580) to develop perianal abscess. In contrast female RR was only 0.81 (95% CI 0.5724 to 1.152).

Factors such as diet and physical activity may play a role in the development of the disease as is strongly suggested by the difference in incidence rates between Jews and Arabs living in the same region. The traditional diet of Arab populations in Israel is richer in high residual elements than that of the Jews [7]. Arabs also engage in more extensive physical activity, a factor well recognized to affect the incidence of some diseases. Different dietary patterns of the Jewish and Arab population may play a role in the relatively higher occurrence of perianal abscess among Bedouin males. However, dietary differences are unlikely to be responsible for all the findings, since the relative risk among Bedouin female was the same as in the general population.

The initial approach to anorectal abscess is incision and drainage (ID). This is a simple procedure which can be performed by residents with a high rate of success and a low rate of complications. However, ID carries with it an increased risk of recurrence. To avoid recurrence, fistulotomy which is directed toward the pathophysiological process of abscess formation should be considered instead of incision and drainage. Nevertheless, fistulotomy is a much more complicated procedure than ID, should be performed by a trained proctologist, and carries a higher rate of morbidity.

In our study, patients were treated by ID; 16.6% of the patients experienced recurrence, a far lower rate than observed in previous reports [8]. Since Soroka Medical Center is the only referral hospital in southern Israel it is reasonable that we would encounter the entire population of patients, including those who experience recurrence. Therefore, we conclude that in our general population it is not justified to perform more than ID during the acute phase.

## Conclusions

The reason for the significant occurrence differences between Bedouin males and the general population is not completely clear and needs additional research. Nevertheless the present observational study has enabled us to isolate certain subpopulations that may require a different management approach. Bedouin males have a high RR for recurrence, as do non-Bedouin who have had a first recurrence (RR 1.8, 2 respectively). For both groups of patients, the evidence indicates that combined operations: Drainage and fistulotomy by experienced proctologist are justified on the grounds that these will minimize the recurrence of perianal abscess.

## Competing interests

There are no competing interests. No grant or support of any kind was received for this manuscript.

## Authors’ contributions

Each author contributed substantially to manuscript. DC: Substantial contributions to conception and design, acquisition of data, analysis and interpretation of data; b. writing the original manuscript. GS: Contributions to conception and design b. drafting the article. II: Contributions to analysis and interpretation of data; b. drafting the article. IP: Contributions to interpretation of data; b. drafting the article. JP: Contributions to interpretation of data; b. drafting the article and revising it critically for important intellectual content. GS: Contributions to interpretation of data; b. Drafting the article and revising it critically for important intellectual content. All authors read and approved the final manuscript.

## Pre-publication history

The pre-publication history for this paper can be accessed here:

http://www.biomedcentral.com/1471-2482/13/35/prepub
